# Development of predictive risk models for major adverse cardiovascular events among patients with type 2 diabetes mellitus using health insurance claims data

**DOI:** 10.1186/s12933-018-0759-z

**Published:** 2018-08-24

**Authors:** James B. Young, Marjolaine Gauthier-Loiselle, Robert A. Bailey, Ameur M. Manceur, Patrick Lefebvre, Morris Greenberg, Marie-Hélène Lafeuille, Mei Sheng Duh, Brahim Bookhart, Carol H. Wysham

**Affiliations:** 1 Cleveland Clinic Foundation Lerner College of Medicine of Case Western Reserve University, Cleveland, OH USA; 2 Analysis Group, Inc., 1000 De La Gauchetière Ouest, Suite 1200, Montreal, QC H3B 4W5 Canada; 3 Janssen Scientific Affairs, LLC, Raritan, NJ USA; 4 Analysis Group Inc., Boston, MA USA; 5 Rockwood Clinic, Spokane, WA USA

**Keywords:** Type 2 diabetes mellitus, Cardiovascular disease, Risk prediction, Health insurance claims

## Abstract

**Background:**

There exist several predictive risk models for cardiovascular disease (CVD), including some developed specifically for patients with type 2 diabetes mellitus (T2DM). However, the models developed for a diabetic population are based on information derived from medical records or laboratory results, which are not typically available to entities like payers or quality of care organizations. The objective of this study is to develop and validate models predicting the risk of cardiovascular events in patients with T2DM based on medical insurance claims data.

**Methods:**

Patients with T2DM aged 50 years or older were identified from the Optum™ Integrated Real World Evidence Electronic Health Records and Claims de-identified database (10/01/2006–09/30/2016). Risk factors were assessed over a 12-month baseline period and cardiovascular events were monitored from the end of the baseline period until end of data availability, continuous enrollment, or death. Risk models were developed using logistic regressions separately for patients with and without prior CVD, and for each outcome: (1) major adverse cardiovascular events (MACE; i.e., non-fatal myocardial infarction, non-fatal stroke, CVD-related death); (2) any MACE, hospitalization for unstable angina, or hospitalization for congestive heart failure; (3) CVD-related death. Models were developed and validated on 70% and 30% of the sample, respectively. Model performance was assessed using C-statistics.

**Results:**

A total of 181,619 patients were identified, including 136,544 (75.2%) without prior CVD and 45,075 (24.8%) with a history of CVD. Age, diabetes-related hospitalizations, prior CVD diagnoses and chronic pulmonary disease were the most important predictors across all models. C-statistics ranged from 0.70 to 0.81, indicating that the models performed well. The additional inclusion of risk factors derived from pharmacy claims (e.g., use of antihypertensive, and use of antihyperglycemic) or from medical records and laboratory measures (e.g., hemoglobin A1c, urine albumin to creatinine ratio) only marginally improved the performance of the models.

**Conclusion:**

The claims-based models developed could reliably predict the risk of cardiovascular events in T2DM patients, without requiring pharmacy claims or laboratory measures. These models could be relevant for providers and payers and help implement approaches to prevent cardiovascular events in high-risk diabetic patients.

**Electronic supplementary material:**

The online version of this article (10.1186/s12933-018-0759-z) contains supplementary material, which is available to authorized users.

## Background

Type 2 diabetes may cause complications of microvascular origin, including nephropathy, neuropathy, and retinopathy, or macrovascular origin, including peripheral artery disease and cardiovascular disease (CVD) [1, 2]. Although diabetes clinical practice guidelines are intended to reflect consensus and evidence-based best medical practices, different entities have some conflicting recommendations, and providing high-quality and detailed guidelines for specific patient subgroups remains challenging [3]. For example, relative to non-diabetic patients, patients with type 2 diabetes have a two- to threefold higher risk of suffering from a CVD event, including a higher risk of myocardial infarction (MI), stroke, unstable angina, and congestive heart failure [4–7], and a higher rate of CVD-related death [8]. Therefore, certain patients with type 2 diabetes could benefit from specialized care that both improve glycemic control and mitigate the risk of CVD.

Thus, having reliable tools making use of readily available data to predict the risk of cardiovascular events among patients with type 2 diabetes may allow healthcare resources to be directed towards patients at high risk, and help healthcare providers meet new quality standard of care. In fact, in 2016, the National Committee for Quality Assurance (NCQA) implemented a new Healthcare Effectiveness Data and Information Set (HEDIS) performance measure based on the rates of hospitalization for potentially preventable complications [9]. More specifically, this measure, which is used by over 90% of health plans in the US [9], targets, among other complications, diabetes short- and long-term complications, including CVD events leading to hospitalization [10]. This means that higher rates of adverse cardiovascular events among patients with type 2 diabetes may negatively affect the NCQA ratings of healthcare providers. Moreover, given the high costs incurred by patients with both CVD and diabetes [11], using such tool efficiently may translate into significant cost savings.

Several of the predictive CVD risk models that have been developed for the general population include diabetes as a risk factor, with models derived from the Framingham Heart Study being among the most well-known [12–14]. Scores based on the Framingham risk models assign weights to risk factors in order to predict cardiovascular events separately for men and women. Risk factors identified for CVD include older age, smoking status, treated and untreated systolic blood pressure, total cholesterol and high-density-lipoprotein cholesterol levels, and diabetes [12–14]. However, the Framingham risk models were not developed for patients with diabetes, and were shown to systematically underestimate CVD risk in this population [15]. In fact, the characteristics of patients enrolled in the Framingham study may differ from real-world populations with diabetes in several ways, including the proportion of minorities, socioeconomic determinants of health, and comorbidity burden [16]. Thus, other risk models have been developed for this population, but all of them rely on data from medical records [17–23]. For example, risk models derived from the United Kingdom Prospective Diabetes Study (UKPDS) identified several risk factors that cannot be used as quantitative predictors using health insurance claims, such as duration of type 2 diabetes, glycated hemoglobin (HbA1c) levels, systolic blood pressure, and cholesterol/high-density lipoprotein ratio [21, 23]. Similarly, the ADVANCE study identified age at diabetes diagnosis, known duration of diabetes, pulse pressure, treated hypertension, HbA1c, urinary albumin/creatinine ratio, and non-HDL cholesterol among risk factors for CVD events; these risk factors cannot be assessed using health insurance claims [22]. Consequently, these models cannot be used to predict CVD risk by entities, like payers, that do not have access to information derived from medical records or laboratory results.

As the face of healthcare provision changes and population management evolves, entities such as public and private payers are moving toward a capitated system of reimbursement, with payments made based on value rather than volume of care. It is thus important for both payers and providers to be able to assess the risks in a given population. Therefore, a CVD risk assessment tool based solely on accessible medical data such as health insurance claims would be relevant for payers to help identify patients with type 2 diabetes at high risk of CVD events. In fact, rationally allocating resources towards these patients by, for example, including CVD risk models in a tool made available to healthcare providers may result in reduced morbidity, mortality, and cost savings. Thus, this study aimed to develop new predictive models and assess their performance in predicting the risk of cardiovascular events in patients with type 2 diabetes based solely on information available in medical health insurance claims. More specifically, models were developed for patients without prior CVD events (hereinafter referred to as the primary prevention population) and for patients with prior CVD events (hereinafter referred to as the secondary prevention population).

## Methods

### Study design

A retrospective observational study design was used to model the risk of CVD events in patients with type 2 diabetes (Additional file 1). The *index date* was defined as a randomly selected date among those with a diagnosis of type 2 diabetes (International Classification of Diseases, 9th Revision, Clinical Modification [ICD-9-CM]: 250.x0 and 250.x2, International Classification of Diseases, 10th Revision, Clinical Modification [ICD-10-CM]: E11.xxx) followed by ≥ 13 months of continuous healthcare plan enrollment. The random selection enabled us to capture a representative sample of patients from a real-world setting with various disease duration. Risk factors for cardiovascular events were assessed during the *baseline period*, defined as the first 12 months following the index date. Cardiovascular events were monitored during the subsequent *at*-*risk period,* which was required to last ≥ 1 month and spanned from the end of the baseline period until the earliest among (i) end of data availability, (ii) end of continuous healthcare plan enrollment, or (iii) death. For each study outcome, the at-risk period was censored at the first occurrence of a given study outcome (see study outcomes section for more details).

### Data source

The Optum™ Integrated Real-World Evidence Electronic Health Records and Claims database (*Optum database*), which combines de-identified electronic medical records and insurance claims, was used to develop and validate the risk models (October 1, 2006–September 30, 2016). This database comprises information on demographics, medical history, and diagnoses for all types of medical encounters (i.e., intensive care unit, emergency department [ED], ward, etc.), in-hospital procedures and medication administrations, prescriptions, laboratory results, and date of death. The database is de-identified and fully compliant with the patient confidentiality requirements of the Health Insurance Portability and Accountability Act (HIPAA).

### Study population

Patients ≥ 50 years with ≥ 1 recorded diagnosis for type 2 diabetes (i.e., ICD-9-CM: 250.x0, and 250.x2; ICD-10-CM: E11.xxx) were included in the study. Patients were required to have ≥ 13 months of continuous eligibility in their healthcare plan after the index date. Patients were excluded if they had ≥ 1 recorded diagnosis for type 1 or gestational diabetes mellitus (i.e., ICD-9-CM: 250.x1, 250.x3, and 648.8x; ICD-10-CM: E10.xxx, O24.4xx, and O99.81x). Moreover, given the growing evidence suggesting that these medications may mitigate cardiovascular risk, to avoid potential confounding, patients were further excluded if they had ≥ 1 prescription fill for a sodium glucose co-transporter 2 (SGLT2) inhibitor or a glucagon-like peptide-1 (GLP-1) receptor agonist at any time during the study period [24–27].

The study population was further stratified into the primary and secondary prevention populations based on whether patients had ≥ 1 diagnosis for any cardiovascular events of interest (see below) in any setting (i.e., inpatient [IP], ED, or outpatient) prior to the at-risk period.

### Study outcomes

Study outcomes included (1) any major adverse cardiovascular event (MACE), which comprised non-fatal MI, non-fatal stroke, and CVD-related death (defined below), (2) any MACE, hospitalization for unstable angina, or hospitalization for congestive heart failure; hereinafter referred to as MACE-plus, and (3) CVD-related death, defined as a death occurring within 30 days after a diagnosis for MI, stroke, unstable angina, heart failure, sudden cardiac arrest, cardiogenic shock, other cerebrovascular events, or other cardiovascular events recorded in a medical claim in any setting (Additional file 2 for ICD codes).

Of note, because it was not possible to determine whether diagnoses for MI or stroke recorded in outpatient settings were actual cardiovascular events or follow-up visits for which the diagnosis was recorded for billing purposes, only diagnoses recorded in an ED or IP settings were considered in the risk models; diagnoses could be recorded in any position.

### Statistical analyses

Distinct predictive risk models were developed for the primary and secondary prevention populations for each of the three study outcomes. A split sample approach was used: The primary and secondary prevention populations were each randomly split into a training (70% of the sample) and a validation (30% of the sample) set. The training sets were used to develop the predictive models, and the validation sets were used to assess the predictive accuracy of the models.

For the prediction of study outcomes, potential risk factors were derived from the published literature and included age, gender, race, ethnicity, year, region, insurance type, prior cardiovascular events, time since first observed type 2 diabetes diagnosis, number of diabetes-related medical visits, Charlson comorbidity index (CCI) [28], adapted diabetes complications severity index (aDCSI) [29], and recorded diagnosis for selected comorbidities such as hypertension, hyperlipidemia, infections, mental disorders, chronic pulmonary disease, and obesity. Univariate associations between potential risk factors and outcomes were assessed; in order to develop more parsimonious models, risk factors were excluded if the standardized difference between patients with and without a given outcome was below 0.10, or if they were present in less than 0.5% of the sample.

Pooled logistic regression models were developed to relate each candidate risk factor to outcomes at pre-specified time points during the at-risk period. A logistic regression model was selected because it can estimate the probability of an event occurring in an interval of time [30]. More specifically, for each patient, the at-risk period was stratified into windows of 6 months during which the outcomes were assessed. For example, the follow-up of a patient who had MACE 15 months after the beginning of the at-risk period was censored at the occurrence of this outcome and stratified in three windows in the regression model: (1) 0–6 months without MACE, (2) 6–12 months without MACE, and (3) 12–18 months with a MACE. For all windows, risk factors were evaluated at baseline, and indicator variables for each time interval were included in the regression models. The risk factors included in the final risk models were chosen using a stepwise variable selection approach based on Akaike’s Information Criterion, in conjunction with tenfold cross-validation methods within the training set. Further specifications of risk factors were tested and variance inflation factor analysis was used to assess the presence of multicollinearity between risk factors, which resulted in the final models.

The performance of the final risk models was evaluated based on discrimination (i.e., C-statistics) in the training and validation sets [31]. The C-statistic is a measure of the predictive accuracy of a logistic regression, which varies between 0.5 (random discrimination) and 1.0 (perfect discrimination). It corresponds to the area under the receiver operating characteristic (ROC) curve [32]. In order to provide a more comprehensive view of the performance of models based on information derived from medical claims, other models that included risk factors derived from medical claims, pharmacy claims, and medical records and laboratory results were developed.

## Results

**Fig. 1:**
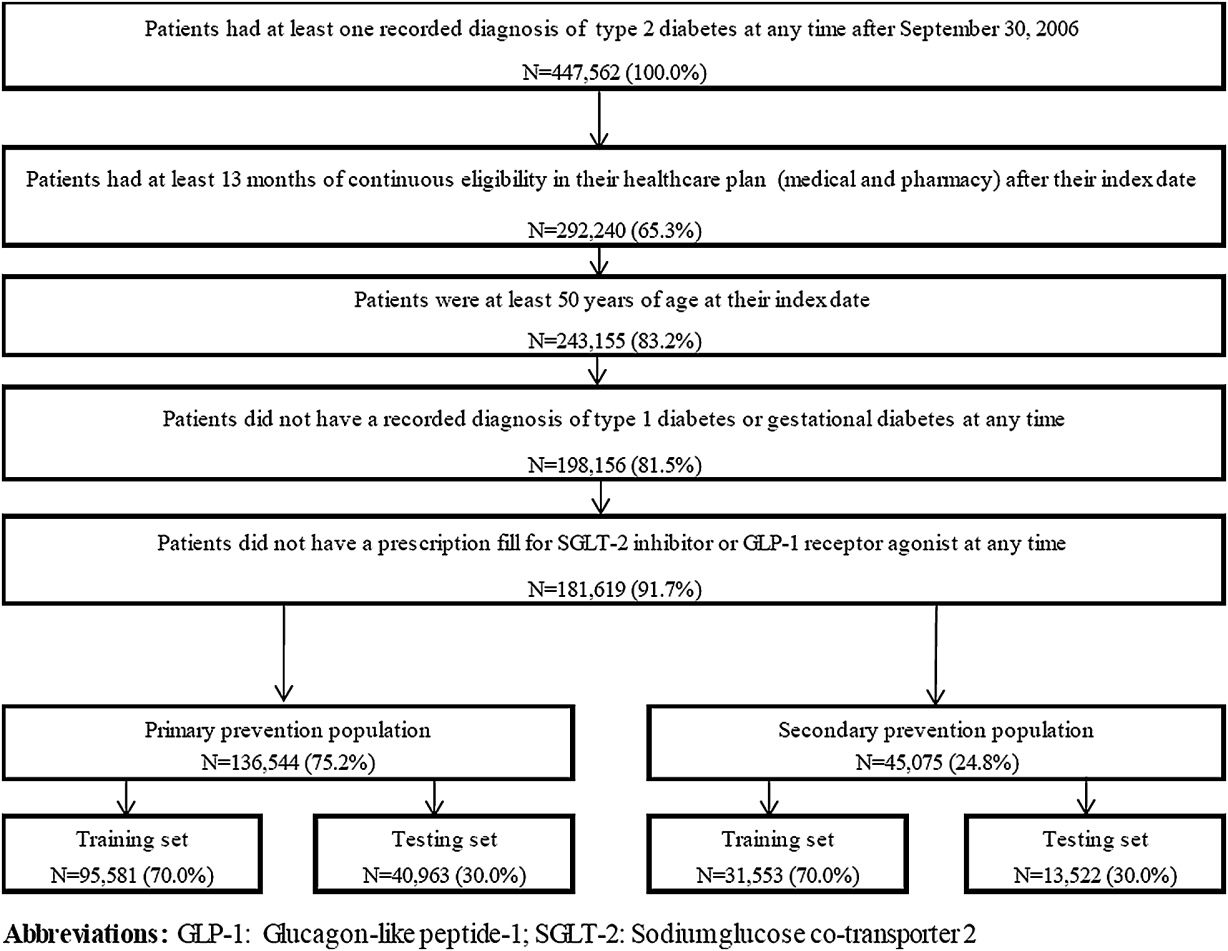
Sample selection

A total of 181,619 patients with type 2 diabetes were included in the study; 136,544 (75.2%) in the primary prevention population and 45,075 (24.8%) in the secondary prevention population (Fig. 1). Among patients in the training set and in the primary prevention population, the proportions of patients with MACE, MACE-plus, and CVD-related death during the at-risk period were 4.7%, 6.5%, and 1.8%, respectively (Additional file 3). In the secondary prevention population, the same proportions were 16.5%, 24.9%, and 8.2%, respectively (Additional file 3). The median duration of the at-risk period following the index date in the training set of the primary prevention population was 12 months (range 1–109 months), with 5.4% of patients having a follow-up longer than 60 months. The median duration of the at-risk period in the training set of the secondary prevention population was 11 months (range 1–108 months), with 3.9% of patients having a follow-up longer than 60 months.

Patients with a CVD event during the at-risk period were older and had higher aDCSI scores compared to patients without CVD events for both the primary and the secondary prevention populations (primary prevention population: mean age = 72.7 vs. 66.4 years, mean aDCSI = 1.9 vs. 1.1, respectively; secondary prevention population: mean age = 75.0 vs. 71.4 years, mean aDCSI = 4.1 vs. 3.2, respectively; Additional file 3). Most patients (> 75%) had a recorded diagnosis for hypertension and/or hyperlipidemia in both the primary and secondary prevention populations. Moreover, compared to patients without CVD events, patients with a CVD event during the at-risk period were more likely to have a recorded diagnosis for select baseline comorbidities—such as infections (primary prevention population: 53.8% vs. 48.8%; secondary prevention population: 69.1% vs. 61.5%, respectively) chronic pulmonary disease (primary prevention population: 22.7% vs. 15.6%; secondary prevention population: 44.5% vs. 31.4%, respectively), and peripheral vascular disorders (primary prevention population: 19.0% vs. 9.3%; secondary prevention population: 34.3% vs. 26.1%, respectively) (Additional file 3).

### Risk models

For the primary prevention population, a total of 12–17 risk factors were included in the models, and most of them were significantly associated with the study outcomes (Table 1). Across all study outcomes, age was the risk factor with the largest impact on the risk of having an event (Table 1). Other risk factors consistently associated with a significantly higher risk of cardiovascular events were recorded diagnosis for other CVD-related conditions (i.e., conditions used to define CVD-related death), diabetes-related hospitalization, higher aDCSI score, recorded diagnosis for chronic pulmonary disease, cancer, fluid and electrolyte disorder, or coagulopathy, and having the baseline period prior to 2011 (Table 1). In addition, hypertension was associated with a higher risk of MACE-plus, while deficiency anemia and pulmonary circulation disorders were associated with a higher risk of CVD-related death (Table 1). Being commercially insured was associated with a lower risk of CVD events for all outcomes, being a female was associated with a lower risk of MACE and CVD-related death, and being Hispanic or Asian was associated with a lower risk of CVD-related death (Table 1).

**Table 1:** Risk models for MACE in the primary prevention population

	MACE		MACE-plus		CVD-related death	
Validation						
C-statistic, training set	0.72		0.71		0.81	
C-statistic, validation set	0.72		0.72		0.81	

Predictors	OR (95% CI)	P-value	OR (95% CI)	P-value	OR (95% CI)	P-value
Age group (reference: 50–54 years old)						
55–59 years old	1.25 (1.04, 1.51)	0.019*	1.19 (1.02, 1.38)	0.028*	1.02 (0.65, 1.59)	0.943
60–64 years old	1.70 (1.43, 2.04)	< 0.001*	1.59 (1.37, 1.83)	< 0.001*	2.13 (1.43, 3.17)	< 0.001*
65–69 years old	1.70 (1.42, 2.04)	< 0.001*	1.58 (1.36, 1.84)	< 0.001*	2.35 (1.59, 3.49)	< 0.001*
70–74 years old	2.00 (1.66, 2.40)	< 0.001*	1.85 (1.59, 2.16)	< 0.001*	2.92 (1.96, 4.33)	< 0.001*
75–79 years old	2.49 (2.06, 3.00)	< 0.001*	2.30 (1.97, 2.69)	< 0.001*	4.12 (2.77, 6.13)	< 0.001*
80–84 years old	4.15 (3.45, 4.99)	< 0.001*	3.48 (2.98, 4.05)	< 0.001*	7.86 (5.31, 11.64)	< 0.001*
85 and above	4.48 (3.62, 5.55)	< 0.001*	3.88 (3.24, 4.64)	< 0.001*	7.96 (5.18, 12.23)	< 0.001*
Female	0.73 (0.69, 0.78)	< 0.001*			0.70 (0.63, 0.77)	< 0.001*
Race (reference: Caucasian)						
African American					0.97 (0.81, 1.15)	0.708
Asian					0.50 (0.31, 0.81)	0.005*
Other/unknown					0.95 (0.83, 1.10)	0.514
Ethnicity (reference: non-Hispanic)						
Hispanic					0.52 (0.35, 0.78)	0.001*
Unknown					1.29 (1.14, 1.47)	< 0.001*
End of baseline period prior to 2011^a^	1.32 (1.23, 1.41)	< 0.001*	1.35 (1.27, 1.43)	< 0.001*	1.90 (1.71, 2.12)	< 0.001*
Geographic region (reference: South)						
Midwest					0.84 (0.75, 0.95)	0.004*
Northeast					0.57 (0.48, 0.68)	< 0.001*
West					0.77 (0.65, 0.91)	0.003*
Other/unknown					0.89 (0.66, 1.21)	0.471
Insurance type (reference: health maintenance organization [HMO])						
Point-of-service (POS)	1.20 (1.03, 1.39)	0.020*	1.23 (1.09, 1.40)	0.001*	1.03 (0.75, 1.41)	0.841
Preferred provider organization (PPO)	1.09 (0.95, 1.24)	0.210	1.17 (1.05, 1.31)	0.006*	1.09 (0.88, 1.35)	0.450
Exclusive provider organization (EPO)	1.34 (1.06, 1.71)	0.015*	1.32 (1.08, 1.61)	0.007*	1.00 (0.59, 1.70)	0.996
Indemnity (IND)	1.71 (1.38, 2.13)	< 0.001*	1.78 (1.48, 2.13)	< 0.001*	2.01 (1.40, 2.88)	< 0.001*
Other	0.90 (0.84, 0.97)	0.005*	0.94 (0.88, 1.00)	0.038*	0.95 (0.84, 1.07)	0.369
Payer type						
Commercial	0.53 (0.46, 0.61)	< 0.001*	0.58 (0.51, 0.65)	< 0.001*	0.46 (0.34, 0.60)	< 0.001*
Other CVD-related conditions (i.e., conditions used to define CVD-related death)	1.19 (1.11, 1.28)	< 0.001*	1.33 (1.26, 1.41)	< 0.001*	1.10 (0.98, 1.23)	0.116
At least 1 diabetes-related hospitalization	1.36 (1.26, 1.46)	< 0.001*	1.27 (1.19, 1.35)	< 0.001*	1.42 (1.27, 1.59)	< 0.001*
Adapted diabetes complications severity index	1.15 (1.13,1.17)	< 0.001*	1.17 (1.15, 1.19)	< 0.001*	1.19 (1.15, 1.23)	< 0.001*
Recorded diagnoses (ref: no diagnosis)						
Hypertension			1.09 (1.01, 1.18)	0.024*		
Chronic pulmonary disease	1.31 (1.22, 1.41)	< 0.001*	1.36 (1.27, 1.44)	< 0.001*	1.52 (1.35, 1.70)	< 0.001*
Cancer	1.14 (1.05, 1.24)	0.003*	1.12 (1.04, 1.20)	0.003*	1.52 (1.35, 1.72)	< 0.001*
Fluid and electrolyte disorders	1.20 (1.09, 1.32)	< 0.001*	1.21 (1.11, 1.31)	< 0.001*	1.22 (1.06, 1.40)	0.007*
Deficiency anemia					1.22 (1.04, 1.43)	0.013*
Coagulopathy	1.45 (1.25, 1.68)	< 0.001*	1.37 (1.20, 1.56)	< 0.001*	1.97 (1.60, 2.41)	< 0.001*
Pulmonary circulation disorders					1.32 (1.03, 1.69)	0.026*
Time interval (reference: 0–6 months)						
6–12 months	1.00 (0.91, 1.09)	0.980	0.97 (0.90, 1.05)	0.501	1.05 (0.90, 1.22)	0.527
12–18 months	1.05 (0.95, 1.15)	0.363	1.02 (0.94, 1.11)	0.564	1.20 (1.02, 1.41)	0.025*
18–24 months	0.96 (0.86, 1.08)	0.528	0.97 (0.88, 1.07)	0.531	1.15 (0.96, 1.38)	0.132
24–30 months	1.06 (0.94, 1.20)	0.361	0.98 (0.88, 1.09)	0.664	1.20 (0.99, 1.47)	0.068
30–36 months	1.02 (0.89, 1.17)	0.785	0.99 (0.88, 1.11)	0.826	1.27 (1.03, 1.57)	0.028*
36–42 months	1.12 (0.96, 1.30)	0.141	1.06 (0.93, 1.21)	0.380	1.29 (1.02, 1.63)	0.035*
42–48 months	0.98 (0.82, 1.17)	0.820	1.00 (0.86, 1.16)	0.970	1.02 (0.77, 1.35)	0.904
48–54 months	1.07 (0.88, 1.29)	0.511	1.00 (0.84, 1.18)	0.971	1.39 (1.06, 1.83)	0.018*
54–60 months	1.17 (0.95, 1.43)	0.138	1.00 (0.83, 1.21)	0.979	1.18 (0.85, 1.63)	0.328
Over 60 months	1.02 (0.88, 1.18)	0.828	0.97 (0.86, 1.11)	0.693	0.93 (0.74, 1.18)	0.570

*CVD* cardiovascular disease, *MACE* major adverse cardiovascular events, *OR* odds ratio, *CI* confidence interval

* Indicates statistical significance at the 5% level

^a^A glycated hemoglobin threshold (i.e., < 8%) was added to Healthcare Effectiveness Data and Information Set (HEDIS) measure for Comprehensive Diabetes Care in 2009 and to the Diabetes Recognition Program of the National Committee for Quality Assurance in 2010, which may have impacted CVD risk in patients with diabetes (See [43])

For the secondary prevention population, 15–20 risk factors were included in the models, and most of them were significantly associated with the study outcomes (Table 2). As for the primary prevention population, older age was the risk factor with the largest impact on the risk of CVD (Table 2). Diabetes-related hospitalization, higher aDCSI score, recorded diagnosis for chronic pulmonary disease or fluid and electrolyte disorders, and having the baseline period prior to 2011 were consistently associated with a significantly higher risk of CVD events (Table 2). In addition, payer type, time since last recorded CVD diagnosis, prior recorded diagnosis for congestive heart failure or iron-deficiency anemia, and ethnicity were identified as predictors of CVD events for all outcomes (Table 2). Prior MI, stroke, and other CVD-related conditions were associated with a higher risk of MACE and MACE-plus, but not of CVD-related death (Table 2). Other risk factors identified for only certain outcomes included race, region, insurance type, recorded diagnosis for mental disorders, obesity, cancer, peripheral vascular disorders, erectile dysfunction, coagulopathy, and pulmonary circulation disorders (Table 2). Interestingly, while being a female was associated with a lower risk of MACE and CVD-related death in the primary prevention population, gender was not associated with an improved predictive accuracy in the secondary prevention population, and thus, was not included as a risk factor in these models (Table 2). Conversely, obesity was not selected as a risk factor in the primary prevention population, whereas it was associated with a lower risk of MACE and CVD-related death in the secondary prevention population.

**Table 2:** Risk models for MACE in the secondary prevention population

	MACE		MACE-plus		CVD-related death	
Validation						
C-statistic, training set	0.70		0.72		0.78	
C-statistic, validation set	0.70		0.72		0.77	

Predictors	OR (95% CI)	P-value	OR (95% CI)	P-value	OR (95% CI)	P-value
Age group (reference: 50–54 years old)						
55–59 years old	1.08 (0.86, 1.36)	0.513	1.01 (0.85, 1.21)	0.883	1.33 (0.84, 2.11)	0.230
60–64 years old	1.24 (1.00, 1.54)	0.049*	1.12 (0.95, 1.33)	0.168	1.72 (1.11, 2.66)	0.014*
65–69 years old	1.35 (1.10, 1.67)	0.005*	1.20 (1.01, 1.41)	0.033*	2.15 (1.41, 3.28)	< 0.001*
70–74 years old	1.52 (1.24, 1.88)	< 0.001*	1.30 (1.11, 1.54)	0.001*	2.67 (1.76, 4.06)	< 0.001*
75–79 years old	1.72 (1.40, 2.12)	< 0.001*	1.43 (1.21, 1.68)	< 0.001*	3.13 (2.06, 4.75)	< 0.001*
80–84 years old	2.20 (1.79, 2.70)	< 0.001*	1.82 (1.55, 2.14)	< 0.001*	4.60 (3.04, 6.97)	< 0.001*
85 and above	2.23 (1.79, 2.77)	< 0.001*	1.81 (1.52, 2.15)	< 0.001*	4.82 (3.14, 7.39)	< 0.001*
Race (reference: Caucasian)						
African American					0.81 (0.70, 0.93)	0.003*
Asian					0.58 (0.37, 0.92)	0.021*
Other/unknown					1.04 (0.93, 1.17)	0.474
Ethnicity (reference: non-Hispanic)						
Hispanic	0.98 (0.82, 1.17)	0.830	0.92 (0.79, 1.08)	0.315	0.69 (0.51, 0.93)	0.016*
Unknown	1.16 (1.10, 1.24)	< 0.001*	1.14 (1.09, 1.20)	< 0.001*	1.14 (1.03, 1.27)	0.013*
End of baseline period prior to 2011^a^	1.43 (1.34, 1.53)	< 0.001*	1.39 (1.32, 1.48)	< 0.001*	1.83 (1.67, 2.01)	< 0.001*
Geographic region (reference: South)						
Midwest					0.83 (0.75, 0.91)	< 0.001*
Northeast					0.73 (0.63, 0.83)	< 0.001*
West					0.84 (0.73, 0.97)	0.014*
Other/unknown					1.07 (0.85, 1.33)	0.570
Insurance type (reference: health maintenance organization [HMO])						
Point-of-service (POS)			1.11 (0.97, 1.27)	0.142	0.79 (0.60, 1.05)	0.105
Preferred provider organization (PPO)			1.21 (1.08, 1.34)	< 0.001*	0.84 (0.69, 1.03)	0.092
Exclusive provider organization (EPO)			1.08 (0.86, 1.36)	0.483	0.58 (0.34, 0.98)	0.043*
Indemnity (IND)			1.29 (1.07, 1.55)	0.007*	1.23 (0.91, 1.66)	0.183
Other			1.01 (0.96, 1.07)	0.631	0.89 (0.81, 0.98)	0.018*
Payer type						
Commercial	0.81 (0.75, 0.89)	< 0.001*	0.77 (0.68, 0.87)	< 0.001*	0.84 (0.67, 1.06)	0.135
Prior CVD diagnoses (ref: no diagnosis)						
Myocardial infarction	1.24 (1.16, 1.33)	< 0.001*	1.13 (1.07, 1.20)	< 0.001*		
Stroke	1.34 (1.26, 1.43)	< 0.001*	1.13 (1.06, 1.19)	< 0.001*		
Congestive heart failure	1.39 (1.30, 1.49)	< 0.001*	1.91 (1.80, 2.03)	< 0.001*	1.97 (1.78, 2.18)	< 0.001*
Time from last observed CVD diagnosis to end of baseline period, months (reference: less than 1 month)						
1–3 months	0.80 (0.73, 0.87)	< 0.001*	0.76 (0.70, 0.82)	< 0.001*	0.83 (0.74, 0.93)	0.002*
3–6 months	0.66 (0.60, 0.73)	< 0.001*	0.62 (0.57, 0.67)	< 0.001*	0.63 (0.56, 0.72)	< 0.001*
6–12 months	0.58 (0.53, 0.63)	< 0.001*	0.52 (0.48, 0.56)	< 0.001*	0.51 (0.45, 0.58)	< 0.001*
12 months and above	0.56 (0.51, 0.61)	< 0.001*	0.48 (0.45, 0.52)	< 0.001*	0.48 (0.42, 0.54)	< 0.001*
Other CVD-related conditions (i.e., Conditions used to define CVD-related death)	1.10 (1.00, 1.20)	0.043*	1.18 (1.09, 1.27)	< 0.001*		
At least 1 diabetes-related hospitalization	1.30 (1.23, 1.39)	< 0.001*	1.27 (1.20, 1.33)	< 0.001*	1.36 (1.25, 1.49)	< 0.001*
Adapted diabetes complications severity index	1.10 (1.08, 1.11)	< 0.001*	1.09 (1.07, 1.11)	< 0.001*	1.11 (1.09, 1.13)	< 0.001*
Recorded diagnoses (ref: no diagnosis)						
Mental disorders					1.14 (1.05, 1.24)	0.002*
Chronic pulmonary disease	1.18 (1.11, 1.25)	< 0.001*	1.27 (1.21, 1.34)	< 0.001*	1.28 (1.18, 1.40)	< 0.001*
Obesity	0.81 (0.74, 0.89)	< 0.001*			0.78 (0.68, 0.88)	< 0.001*
Cancer					1.16 (1.05, 1.29)	0.005*
Peripheral vascular disorders			1.07 (1.01, 1.13)	0.017*		
Fluid and electrolyte disorders	1.16 (1.09, 1.24)	< 0.001*	1.14 (1.08, 1.21)	< 0.001*	1.15 (1.05, 1.26)	0.002*
Deficiency anemia	1.19 (1.10, 1.28)	< 0.001*	1.15 (1.08, 1.23)	< 0.001*	1.22 (1.10, 1.35)	< 0.001*
Erectile dysfunction, organic origin					0.60 (0.43, 0.84)	0.003*
Coagulopathy			1.14 (1.05, 1.24)	0.003*	1.17 (1.02, 1.33)	0.024*
Pulmonary circulation disorders			1.21 (1.12, 1.30)	< 0.001*	1.33 (1.18, 1.49)	< 0.001*
Time interval (reference: 0–6 months)						
6–12 months	0.82 (0.76, 0.89)	< 0.001*	0.78 (0.73, 0.83)	< 0.001*	0.89 (0.79, 0.99)	0.030*
12–18 months	0.77 (0.70, 0.84)	< 0.001*	0.74 (0.69, 0.80)	< 0.001*	0.81 (0.72, 0.93)	0.002*
18–24 months	0.80 (0.72, 0.89)	< 0.001*	0.70 (0.64, 0.77)	< 0.001*	0.82 (0.71, 0.95)	0.009*
24–30 months	0.71 (0.62, 0.80)	< 0.001*	0.58 (0.52, 0.66)	< 0.001*	0.78 (0.65, 0.92)	0.004*
30–36 months	0.67 (0.58, 0.78)	< 0.001*	0.57 (0.50, 0.66)	< 0.001*	0.79 (0.65, 0.96)	0.019*
36–42 months	0.71 (0.59, 0.84)	< 0.001*	0.63 (0.54, 0.74)	< 0.001*	0.73 (0.58, 0.93)	0.009*
42–48 months	0.77 (0.63, 0.93)	0.007*	0.70 (0.59, 0.83)	< 0.001*	0.77 (0.59, 1.00)	0.052
48–54 months	0.61 (0.47, 0.78)	< 0.001*	0.50 (0.39, 0.63)	< 0.001*	0.66 (0.48, 0.90)	0.010*
54–60 months	0.69 (0.53, 0.91)	0.007*	0.63 (0.49, 0.81)	< 0.001*	0.75 (0.53, 1.05)	0.097
Over 60 months	0.56 (0.46, 0.69)	< 0.001*	0.50 (0.41, 0.61)	< 0.001*	0.51 (0.38, 0.67)	< 0.001*

*CVD* cardiovascular disease, *MACE* major adverse cardiovascular events, *OR* odds ratio, *CI* confidence interval

* Indicates statistical significance at the 5% level

^a^A glycated hemoglobin threshold (i.e., < 8%) was added to Healthcare Effectiveness Data and Information Set (HEDIS) measure for Comprehensive Diabetes Care in 2009 and to the Diabetes Recognition Program of the National Committee for Quality Assurance in 2010, which may have impacted CVD risk in patients with diabetes (See [43])

**Fig. 2:**
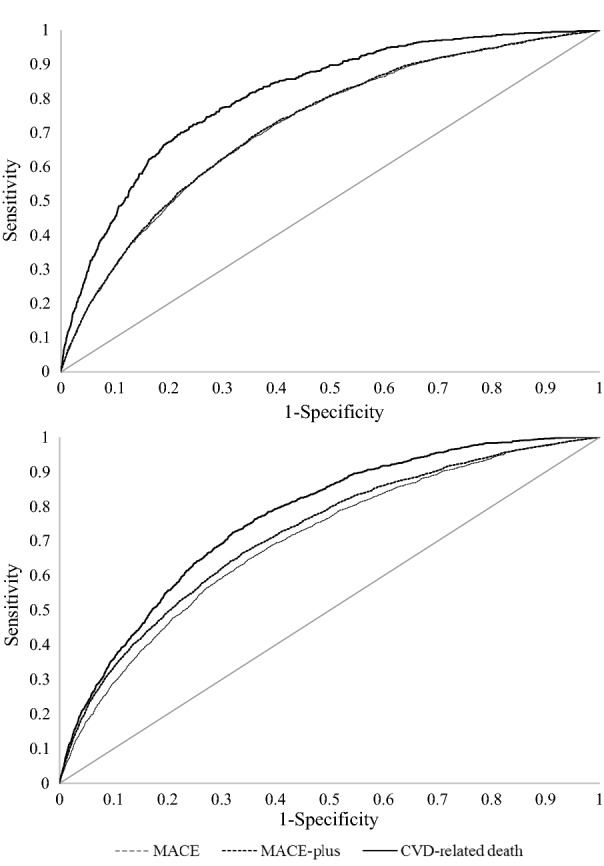
Receiver operating characteristic curves for the risk models. **a** Primary prevention population, testing set. **b** Secondary prevention population, testing set

The risk models performed well in predicting MACE, MACE-plus, and CVD-related death with C-statistics ranging between 0.70 and 0.81 when considering both the training and validation sets (Tables 1 and 2, Fig. 2). Notably, the highest predictive accuracy was observed for models predicting CVD-related death (Tables 1 and 2; Fig. 2). In addition, the models were well calibrated, with differences between the median predicted risk and median observed risk that did not exceed 0.1% for each of the study outcomes in both the primary and secondary prevention populations (data not shown).

In addition, to further assess the potential impact of using information exclusively derived from medical claims data on performance, predictive models that also included risk factors obtained from pharmacy claims, as well as from medical records and laboratory results were developed. These models included up to 11 additional risk factors, but only showed limited improvements in terms of predictive accuracy, with C-statistics increasing by no more than 0.01 in the training and validation sets for both the primary and secondary prevention populations (data not shown).

### Examples

Notably, the risk models can be used to assess CVD risk at different time windows separated by intervals of 6 months over a maximum of 5 years. For instance, the average patient in the primary prevention population—a 67 year old female with an aDCSI score of 1 and recorded diagnosis for hypertension and hyperlipidemia—had a predicted risk of MACE of 1.4% after 1 year, 2.7% after 2 years, and 6.8% after 5 years. The predicted 5-year risk for MACE-plus and CVD-related death were 10.6% and 1.7%, respectively (Table 3: Case 1). For the secondary prevention population, the average patient was a 73 year old male diagnosed with prior congestive heart failure ≥ 12 months ago, other CVD-related conditions, an aDCSI score of 3, recorded diagnosis for hypertension, hyperlipidemia, and infection within the last year. The predicted risk of MACE for that patient were 5.8% after 1 year, 10.5% after 2 years, and 21.8% after 5 years. The predicted 5-year risk for MACE-plus and CVD-related death were 35.2% and 9.9%, respectively (Table 3: Case 2).

**Table 3:** Predicted risk for the average patient in primary and secondary prevention population

Patient characteristics	Case 1			Case 2		
Age	67			73		
Gender	Female			Male		
Type of insurance	Medicare, other			Medicare, other		
Race/ethnicity	White, non-Hispanic			White, non-Hispanic		
Region	Mid-West			South		
Prior CVD diagnoses	None			CHF (≥ 12 months ago), Other CVD-related condition		
aDCSI	1			3		
Diabetes-related hospitalizations	No			No		
Other recorded diagnoses	Hypertension, hyperlipidemia			Hypertension, hyperlipidemia, infection		

Predicted risk^a^	MACE (%)	MACE-plus (%)	CVD-related death (%)	MACE (%)	MACE-plus (%)	CVD-related death (%)
At 6 month	0.7	1.1	0.2	3.2	6.1	1.3
At 1 year	1.4	2.2	0.3	5.8	10.6	2.4
At 2 years	2.7	4.3	0.7	10.5	18.5	4.5
At 3 years	4.1	6.4	1.0	14.4	24.3	6.4
At 5 years	6.8	10.6	1.7	21.8	35.2	9.9

*aDCSI* adapted diabetes complications severity index, *CHF* congestive heart failure, *CVD* cardiovascular disease, *MACE* major adverse cardiovascular event, *CI* confidence interval

^a^Predicted risk for a hypothetical patient based on listed characteristics

## Discussion

This study developed and validated models that predict the risk of adverse cardiovascular events in patients with type 2 diabetes using exclusively information derived from health insurance claims. The main risk factors identified in the primary prevention population included age, diabetes-related hospitalizations, and recorded diagnosis for coagulopathy and chronic pulmonary disease. In the secondary prevention population, age, prior CVD diagnoses, diabetes-related hospitalizations, and recorded diagnosis for chronic pulmonary disease had the most important impact on the risk of having a CVD event. Overall, the models reliably predicted the cardiovascular events for the primary and secondary prevention populations, as illustrated by the C-statistics ranging between 0.70 and 0.81.

The finding that age was one of the most important risk factor in predicting cardiovascular events is consistent with findings in previous studies that primarily focused on a diabetes population, such as the UKPDS risk engine [21, 23] and studies that focused on a general population, such as the Framingham Heart Study [12]. However, a major difference between the models developed in the current study and previous ones is that the latter included risk factors derived from laboratory results and medical records [12, 17, 18, 20–22], which are often not available to national quality of care organizations and payers. In contrast, the current study used only information that is readily available from medical claims data.

Nonetheless, claims-based information can be used as a proxy for risk factors derived from laboratory results and medical records. For example, blood pressure measurements were not available in claims data, but hypertension-identified based on a recorded diagnosis in a medical claim—was included in the models. Similarly, recorded diagnosis for hyperlipidemia was used as a proxy for high-density lipoprotein cholesterol and low-density lipoprotein cholesterol levels, although it was not included in any models. Yet, certain risk factors identified in the Framingham and UKPDS models tend to be underreported in medical claims, and thus, may have limited predictive accuracy in claims-based models. For example, although diagnosis codes for smoking do exist, this condition is typically underreported in medical claims. Therefore, smoking was not included in any of the claims-based models. However because our study was limited to risk factors available in insurance claims data, certain risk factors identified in other studies were not available for selection in the models. In particular, several studies pointed to a link between glycemic markers and CVD [33–37], but given that HbA1c measures are not available in insurance claims data, this potential risk factor could not be included in the models.

This study also found that obesity was associated with a lower risk of MACE and CVD-related death in the secondary prevention population. Several previous studies found obesity to be associated with better survival in patients with chronic or cardiac diseases, hence the term “obesity paradox” to describe this counterintuitive phenomenon [38]. Several explanations have been proposed, including the advantages of fat reserves during illness, biases or confounding in observational studies (e.g., more intensive management), or weight loss due to illness in the reference group [39]. However, due to the observational nature of the current study, no causal relationship can be inferred.

Regardless of the aforementioned differences in the risk factors identified in the current study versus previously published models, the models developed here performed well in predicting the risk of cardiovascular events in a population with two well-defined risk factors, namely patients with type 2 diabetes and above 50 years of age. Overall, the predictive accuracies of the models presented in the current study are comparable to those of previously published models. For example, the Framingham risk score, which included diabetes as a predictor, yielded C-statistics of 0.76 and 0.79 for men and women in the general population, respectively [12]. However, when evaluated in an older diabetic cohort and in patients without prior CVD, the Framingham risk score had a C-statistic of 0.65 [19]. The performances of the claims-based models presented here were also comparable to those of previously developed risk models specific to the diabetic population, such as the UKPDS risk engine [21, 40]. Although C-statistics were not reported in the UKPDS original publications, subsequent validations in other diabetic cohorts yielded C-statistics ranging from 0.61 to 0.73 [19, 41]. The ADVANCE model, developed in a population of diabetic patients at risk of cardiovascular events similar to the secondary prevention population in this study, also presented comparable C-statistics of 0.69-0.70 [22]. Moreover, several other multivariate risk models were published and reported C-statistics ranging between 0.64 and 0.70 [17, 18, 20]. A comprehensive external validation study would be needed to evaluate the performance of the different models on the same cohort of patients [42].

The Framingham and UKPDS models were not developed and tested for patients with a prior history of CVD (i.e., the secondary prevention population), meaning that their predictive accuracy may be lower in this subpopulation [12, 21]. Therefore, another advantage of the models developed in the current study over several previous ones is their ability to predict CVD risk in patients with prior history of CVD, who represented almost a quarter of the sample population. More generally, the reliability of this claims-based approach is perhaps best illustrated by the limited incremental predictive accuracy conferred by the additional inclusion of variables derived from medical records or laboratory results.

In light of the HEDIS performance measure that targets hospitalization for potentially preventable complications, rationally allocating healthcare resources to patients with type 2 diabetes at higher risk of cardiovascular complications may help healthcare providers meet quality of care standards, and lead to reductions in morbidity, mortality, and cost savings. With growing evidence suggesting that certain types of diabetes treatments—such as SGLT2 inhibitors or GLP-1 receptor agonists—may mitigate cardiovascular risk in addition to improving glycemic control, the potential dual purpose of these diabetes medications could be considered-despite their higher cost—to optimize treatment decisions in patients with type 2 diabetes at high risk of CVD [24–27]. Patients receiving these game-changing treatments were excluded from the present study due to the potential for indication bias: the use of SGLT2 inhibitors or GLP1 receptor agonists could effectively reduce the risk of CVD, but may appear as risk factors associated with a higher risk of CVD if these agents are preferentially prescribed to higher-risk patients. Such counterintuitive phenomena are common in observational studies. Another potential clinical application of the models developed here would be to identify patients at high risk of CVD events within a certain time window in order to provide preventive care. The threshold used for this high-risk group could be rationally determined using the risk that maximizes the sum of the model sensitivity and specificity. For example, using this method, the high-risk threshold in the primary prevention population would be 2.5%, 3.5%, and 1.0% for MACE, MACE-plus, and CVD-related death, respectively (sensitivity ranging from 67 to 73%, and specificity ranging from 67 to 76%). In the sample population used in the current study, applying these thresholds would result in approximately one out of three patients classified at high-risk of having MACE or MACE-plus within a 1-year window, and one out of four patients at high-risk of CVD-related death. In the secondary prevention population, the same thresholds would be 12.5%, 18.0%, and 5.0% for MACE, MACE-plus, and CVD-related death, respectively (sensitivity ranging from 62 to 78%, and specificity ranging from 66 to 72%), resulting in approximately one out of three patients with a high-risk of having any cardiovascular event within a 1-year window.

### Limitations

The current study is subject to a few limitations. First, the identification of study outcomes was based on definitional algorithms using health insurance claims data that have not been fully validated, which could lead to the misclassification of outcomes. Second, patients may have experienced cardiovascular events prior to the start of data availability, and may have been misclassified in the primary prevention population. Third, a recorded diagnosis code on a medical claim is not an attestation that the patient has the condition, because the code may represent a rule-out diagnosis or may be recorded incorrectly. Fourth, risk predictions beyond 60 months post-index should be interpreted with caution as a limited number of patients had an at-risk period of such duration. Moreover, risk predictions over longer periods may be confounded by changes in therapeutic strategies. Despite these limitations, healthcare claims are a valuable resource to develop such models. Indeed, the large sample size typically available in claims database prevents over-fitting the models to a specific data set, thereby increasing their external validity, as illustrated by the negligible decrease in predictive accuracy observed within the validation set compared to the training set. Future studies are needed to externally validate the model in a distinct population or database. Finally, it should be noted that the risk models developed aimed at identifying patients at risk of CVD events, no causal inference can be drawn from this model based on observational data.

## Conclusions

In summary, this study developed risk models that could reliably identify patients with type 2 diabetes at risk of MACE, MACE-plus, and CVD-related death based on information available in health insurance claims. Ultimately, stakeholders—such as quality of care organizations and payers—may use these models to identify diabetic patients at high risk of cardiovascular events and potentially improve their clinical management, thereby preventing a significant part of the disease burden and associated costs.

## Additional files

**Additional file 1.** Study design.

**Additional file 2.** Definition of outcomes and risk factors.

**Additional file 3.** Risk factors among patients with and without any major adverse cardiovascular events during the at-risk period (training set).

## Abbreviations

aDCSI: adapted diabetes complications severity index
CCI: Charlson comorbidity index
CVD: cardiovascular disease
ED: emergency department
GLP-1: glucagon-like peptide-1
HbA1c: glycated hemoglobin
HEDIS: Healthcare Effectiveness Data and Information Set
HIPAA: Health Insurance Portability and Accountability Act
ICD-9-CM: International Classification of Diseases, 9th Revision, Clinical Modification
ICD-10-CM: International Classification of Diseases, 10th Revision, Clinical Modification
IP: inpatient
MACE: major adverse cardiovascular event
MI: myocardial infarction
NCQA: National Committee for Quality Assurance
SGLT2: sodium glucose co-transporter 2
UKPDS: United Kingdom Prospective Diabetes Study
