# Supplementary figures and images for: Development of predictive risk models for major adverse cardiovascular events among patients with type 2 diabetes mellitus using health insurance claims data
Source: Cardiovasc Diabetol. 2018 Aug 24;17:118. doi: 10.1186/s12933-018-0759-z (PMC6109303; doi:10.1186/s12933-018-0759-z)

## Additional File 1. Study Design

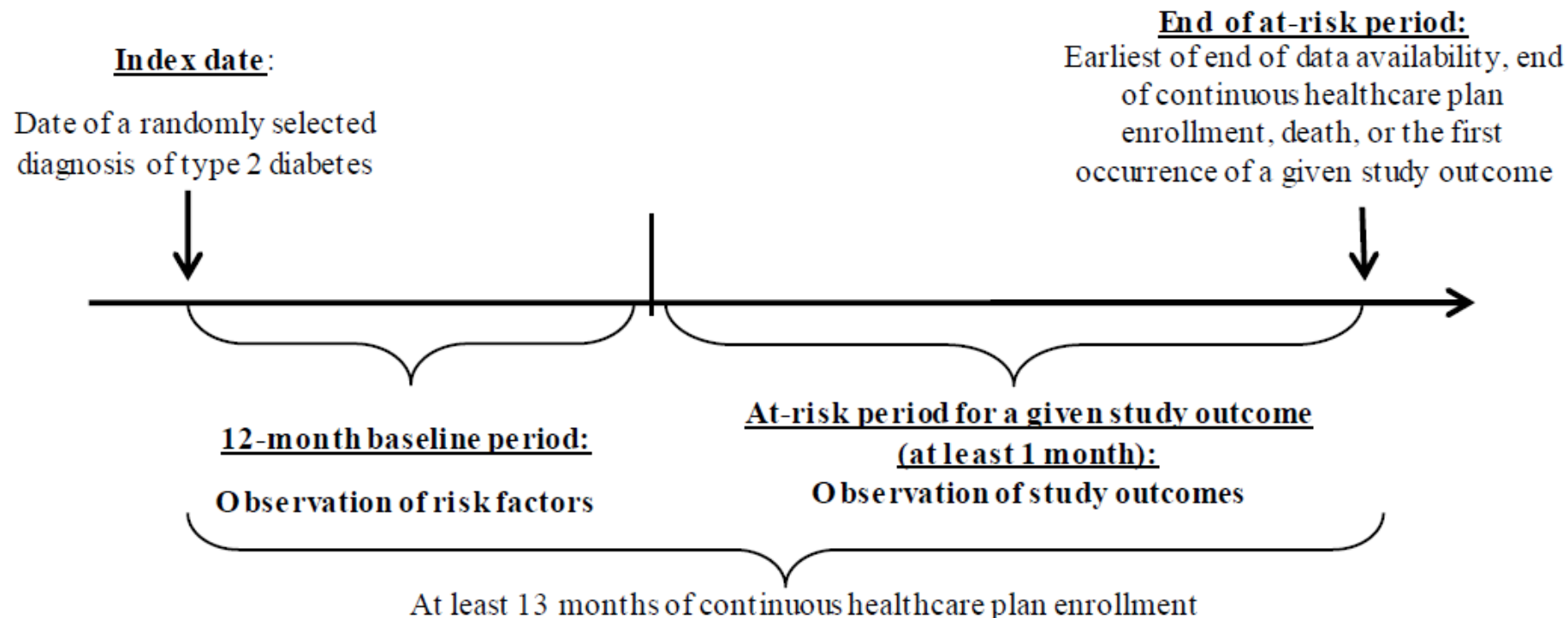

Supplement: Supplementary file 1 — Additional file 1. Study design. [file 12933_2018_759_MOESM1_ESM.pdf]
