# Supplementary material for: Development of predictive risk models for major adverse cardiovascular events among patients with type 2 diabetes mellitus using health insurance claims data
Source: Cardiovasc Diabetol. 2018 Aug 24;17:118. doi: 10.1186/s12933-018-0759-z (PMC6109303; doi:10.1186/s12933-018-0759-z)
Supplement: Supplementary file 2 — Additional file 2. Definition of outcomes and risk factors. [file 12933_2018_759_MOESM2_ESM.docx]

**Additional File 2. Definition of Outcomes and Risk Factors**

|  | **ICD-9-CM** | **ICD-10-CM** |
| --- | --- | --- |
| ***CVD*** |  |  |
| Myocardial infarction | 410.xx | I21.xx, I22.xx |
| Stroke | 430.xx, 431.xx, 434.xx, 436.xx | I60.xx, I61.xx, I63.3-I63.9, I66.xx |
| Unstable angina | 411.1x | I20.0 |
| Congestive heart failure | 428.0x | I50.9 |
| Other CVD-related conditions |  |  |
| Sudden cardiac arrest | 427.5 | I46.9 |
| Heart failure | 428.xx | I50.xx |
| Cardiogenic shock | 785.51 | R57.0 |
| Other cerebrovascular events | 432.xx, 433.xx, 435.xx, 437.xx | I62.xx, I63.0-I63.2, I65.xx, I67.xx, I68.xx |
| Other cardiovascular events | 411.xx-414.xx, 415.xx-417.xx, 420.xx-427.xx, 429.xx | I20.x, I23.xx-I25.xx, I26.xx-I28.xx, I30.xx-I49.x, I51.xx-I52.xx, I64.xx |
| ***Other recorded diagnoses*** |  |  |
| Obesity | 278.xx | E66.x |
| Hypertension | 401.x-405.x | I10.x, I11.x-113.x, I15.x |
| Peripheral vascular disorders | 093.0, 437.3, 440.x, 441.x, 443.1- 443.9, 447.1, 557.1, 557.9, V43.4 | I70.x, I71.x, I73.1, I73.8, 173.9, I77.1, I79.0, I79.2, K55.1, K55.8, K55.9, Z95.8 |
| Pulmonary circulation disorder | 415.0, 415.1, 416.x, 417.0, 417.8, 417.9 | I26.x, 127.x, I28.0, I128.8, I28.9 |
| Chronic pulmonary disease | 416.8, 416.9, 490.x -505.x, 506.4, 508.1, 508.8 | I27.8, I27.9, J40.x-J47.x, J60.x-J67.x, J68.4, J70.1, J70.3 |
| Fluid and electrolyte disorders | 253.6, 276.x | E22.2, E86.x, E87.x |
| Coagulopathy | 286.x, 287.1, 287.3-287.5 | D65-D68.x, D69.1, D69.3-D69.6 |
| Deficiency anemia | 280.1-280.9, 281.x | D50.8, D50.9, D51.x-D53.x |
| Erectile dysfunction, organic origin | 607.84 | N52.9 |
| Cancer | 196.x-199.x, 140.x-172.x, 174.x-195.x, 200.x-202.x, 203.0, 238.6 | C77.x-C80.x, C00.x-C26.x, C30.x-C34.x, C37.x-C41.x, C43.x, C45.x-C58.x, C60.x-C76.x, C97.x, C81.x-C85.x, C88.x, C96.x, C90.0, C90.2 |
| Any mental disorders |  |  |
| Anxiety disorders | 309.21, 312.23, 300.29, 300.23, 300.01, 300.22, 300.02, 293.84, 300.09, 300.00 | F93.0, F94.0, F40.218, F40.228, F40.230, F40.231, F40.232, F40.233, F40.248, F40.298, F40.10, F41.0, F40.00, F41.1, F06.4, F41.8, F41.9 |
| Trauma- and stressor-related disorders | 313.89, 309.81, 308.3x, 309.0x, 309.24, 309.28, 309.3x, 309.4x, 309.9x, 309.89 | F94.1, F94.2, F43.10, F43.0, F43.21, F43.22, F43.23, F43.24, F43.25, F43.20, F43.9, F43.8 |
| Bipolar and related disorders | 296.40-296.46, 296.50-296.56, 296.7x, 296.89, 301.13, 293.83, 296.80 | F31.11,F31.12, F31.13,F31.2, F31.73,F31.74, F31.9,F31.0, F31.31, F31.32,F31.4,F31.5, 31.75, F31.76, F31.9, , F31.81, F31.89, F34.0, F06.33, F06.34, F06.31, F06.32, F31.9 |
| Obsessive-compulsive and related disorders | 300.3x, 312.39, 698.4x, 294.8x | F42, F63.2, L98.1, F06.8 |
| Schizophrenia spectrum and other psychotic disorders | 301.22, 297.1x, 298.8x, 295.40, 295.90, 295.70, 293.81, 293.82, 293.89, 298.8x, 298.9x | F21, F22, F23, F20.81, F20.9, F25.0, F25.1, F06.2, F06.0, F06.1, F28, F29 |
| Depressive disorders | 296.99-296.20-296.26, 296.30-296.36, 300.4x, 625.4x, 311.xx | F34.8, F32.0,F32.1,F32.2, F32.3,F32.4, F32.5,F32.9, F33.0,F33.1 F33.2, F33.3, F33.41, F33.42, F33.9, F34.1, N94.3, F32.8, F32.9 |
| Feeding and eating disorders | 307.52, 307.53, 307.59, 307.1x, 307.51, 307.50 | F98.3, F50.8, F98.21, F50.01, F50.02, F50.2, F50.9 |
| Sleep-wake disorders | 780.52, 780.54, 780.59, 347.00, 347.01, 347.10, 327.23, 327.21, 786.04, 780.57, 327.24, 327.25, 327.26, 307.45, 307.46, 307.47, 327.42, 333.94 | G47.00, G47.09, G47.10, G47.19, G47.8, G47.9, G47.419, G47.411, G47.429, G47.33, G47.31, R06.3, G47.37, G47.34, G47.35, G47.36, G47.21, G47.22, G47.23, G47.24, G47.26, G47.20, F51.3, F51.4, F51.5, G47.52, G25.81 |

**Abbreviations:** CVD: Cardiovascular disease; ICD-9-CM: International Classification of Diseases, 9^th^ Revision, Clinical Modification; ICD-10-CM: International Classification of Diseases, 10^th^ Revision, Clinical Modification; T2DM; Type 2 Diabetes Mellitus
