# Supplementary material for: Development of predictive risk models for major adverse cardiovascular events among patients with type 2 diabetes mellitus using health insurance claims data
Source: Cardiovasc Diabetol. 2018 Aug 24;17:118. doi: 10.1186/s12933-018-0759-z (PMC6109303; doi:10.1186/s12933-018-0759-z)
Supplement: Supplementary file 3 — Additional file 3. Risk factors among patients with and without any major adverse cardiovascular events during the at-risk period (training set). [file 12933_2018_759_MOESM3_ESM.docx]

**Additional File 3. Risk Factors among Patients with and without Any Major Adverse Cardiovascular Events during the At-risk Period (Training Set)**

|  | |  | | | |  | | | |  | |  | |  | | | |  | | | |  | |  |  |  |  |  |  |  |  |  |  |
| --- | --- | --- | --- | --- | --- | --- | --- | --- | --- | --- | --- | --- | --- | --- | --- | --- | --- | --- | --- | --- | --- | --- | --- | --- | --- | --- | --- | --- | --- | --- | --- | --- | --- |
|  | | **Primary CVD prevention population**  **N=95,581** | | | | | | | | | |  | | **Secondary CVD prevention population**  **N=31,553** | | | | | | | | | |  |  |  |  |  |  |  |  |  |  |
|  |  |  |  |  |  |  |  |  |  |  |  |  |  |  | | | | | | | | | |  |  |  |  |  |  |  |  |  |  |
|  | | **With**  **any event^1,2^** | | | | **Without**  **any event** | | | | **Std. diff.** | |  | | **With**  **any event^1,3^** | | | | **Without**  **any event** | | | | **Std. diff.** | |  |  |  |  |  |  |  |  |  |  |
| **Risk factors during the baseline period** | | **N=6,237 (6.5%)** | | | | **N=89,344 (93.5%)** | | | |  |  |  | | **N=7,871 (24.9%)** | | | | **N=23,682 (75.1%)** | | | |  |  |  |  |  |  |  |  |  |  |  |  |
| Age at end of baseline period, mean ± SD [median] | | 72.7 ± 9.1 [73.7] | | | | 66.4 ± 9.5 [66.2] | | | | 0.677 ‡ | |  | | 75.0 ± 8.9 [76.8] | | | | 71.4 ± 10.0 [71.8] | | | | 0.383 ‡ | |  |  |  |  |  |  |  |  |  |  |
| Female | | 2,888 | | (46.3%) | | 45,043 | | (50.4%) | | 0.082 | |  | | 3,737 | | (47.5%) | | 11,123 | | (47.0%) | | 0.010 | |  |  |  |  |  |  |  |  |  |  |
| Ethnicity | |  | |  | |  | |  | |  | |  | |  | |  | |  | |  | |  | |  |  |  |  |  |  |  |  |  |  |
| Not hispanic | | 3,991 | | (64.0%) | | 58,508 | | (65.5%) | | 0.031 | |  | | 4,685 | | (59.5%) | | 15,335 | | (64.8%) | | 0.108 ‡ | |  |  |  |  |  |  |  |  |  |  |
| Hispanic | | 183 | | (2.9%) | | 3,892 | | (4.4%) | | 0.076 | |  | | 193 | | (2.5%) | | 821 | | (3.5%) | | 0.060 | |  |  |  |  |  |  |  |  |  |  |
| Unknown | | 2,063 | | (33.1%) | | 26,944 | | (30.2%) | | 0.063 | |  | | 2,993 | | (38.0%) | | 7,526 | | (31.8%) | | 0.131 ‡ | |  |  |  |  |  |  |  |  |  |  |
| Year at end of baseline period before 2011 | | 2,244 | | (36.0%) | | 20,806 | | (23.3%) | | 0.281 ‡ | |  | | 2,342 | | (29.8%) | | 4,152 | | (17.5%) | | 0.291 ‡ | |  |  |  |  |  |  |  |  |  |  |
| Race | |  | |  | |  | |  | |  | |  | |  | |  | |  | |  | |  | |  |  |  |  |  |  |  |  |  |  |
| Caucasian | | 4,166 | | (66.8%) | | 58,720 | | (65.7%) | | 0.023 | |  | | 4,888 | | (62.1%) | | 15,347 | | (64.8%) | | 0.056 | |  |  |  |  |  |  |  |  |  |  |
| African American | | 585 | | (9.4%) | | 9,244 | | (10.3%) | | 0.032 | |  | | 866 | | (11.0%) | | 2,777 | | (11.7%) | | 0.023 | |  |  |  |  |  |  |  |  |  |  |
| Asian | | 89 | | (1.4%) | | 2,130 | | (2.4%) | | 0.07 | |  | | 95 | | (1.2%) | | 413 | | (1.7%) | | 0.045 | |  |  |  |  |  |  |  |  |  |  |
| Other/unknown | | 1,397 | | (22.4%) | | 19,250 | | (21.5%) | | 0.021 | |  | | 2,022 | | (25.7%) | | 5,145 | | (21.7%) | | 0.093 | |  |  |  |  |  |  |  |  |  |  |
| Geographic region, N (%) | |  | |  | |  | |  | |  | |  | |  | |  | |  | |  | |  | |  |  |  |  |  |  |  |  |  |  |
| Midwest | | 2,363 | | (37.9%) | | 33,903 | | (37.9%) | | 0.001 | |  | | 2,873 | | (36.5%) | | 8,152 | | (34.4%) | | 0.043 | |  |  |  |  |  |  |  |  |  |  |
| South | | 2,354 | | (37.7%) | | 33,819 | | (37.9%) | | 0.002 | |  | | 2,898 | | (36.8%) | | 9,156 | | (38.7%) | | 0.038 | |  |  |  |  |  |  |  |  |  |  |
| Northeast | | 745 | | (11.9%) | | 11,487 | | (12.9%) | | 0.028 | |  | | 1,057 | | (13.4%) | | 3,423 | | (14.5%) | | 0.030 | |  |  |  |  |  |  |  |  |  |  |
| West | | 608 | | (9.7%) | | 7,889 | | (8.8%) | | 0.032 | |  | | 791 | | (10.0%) | | 2,294 | | (9.7%) | | 0.012 | |  |  |  |  |  |  |  |  |  |  |
| Other/Unknown | | 167 | | (2.7%) | | 2,246 | | (2.5%) | | 0.010 | |  | | 252 | | (3.2%) | | 657 | | (2.8%) | | 0.025 | |  |  |  |  |  |  |  |  |  |  |
| Insurance type, N (%) | |  | |  | |  | |  | |  | |  | |  | |  | |  | |  | |  | |  |  |  |  |  |  |  |  |  |  |
| Health maintenance  organization (HMO) | | 2,708 | | (43.4%) | | 25,657 | | (28.7%) | | 0.310 ‡ | |  | | 3,444 | | (43.8%) | | 7,743 | | (32.7%) | | 0.229 ‡ | |  |  |  |  |  |  |  |  |  |  |
| Point-of-service (POS) | | 868 | | (13.9%) | | 27,239 | | (30.5%) | | 0.407 ‡ | |  | | 671 | | (8.5%) | | 3,997 | | (16.9%) | | 0.253 ‡ | |  |  |  |  |  |  |  |  |  |  |
| Preferred provider  organization (PPO) | | 376 | | (6.0%) | | 4,249 | | (4.8%) | | 0.056 | |  | | 462 | | (5.9%) | | 1,258 | | (5.3%) | | 0.024 | |  |  |  |  |  |  |  |  |  |  |
| Exclusive provider  organization (EPO) | | 138 | | (2.2%) | | 4,093 | | (4.6%) | | 0.131 ‡ | |  | | 109 | | (1.4%) | | 643 | | (2.7%) | | 0.094 | |  |  |  |  |  |  |  |  |  |  |
| Indemnity (IND) | | 223 | | (3.6%) | | 1,228 | | (1.4%) | | 0.142 ‡ | |  | | 230 | | (2.9%) | | 462 | | (2.0%) | | 0.063 | |  |  |  |  |  |  |  |  |  |  |
| Other | | 1,924 | | (30.8%) | | 26,878 | | (30.1%) | | 0.017 | |  | | 2,955 | | (37.5%) | | 9,579 | | (40.4%) | | 0.060 | |  |  |  |  |  |  |  |  |  |  |
| Payer type, N(%) | |  | |  | |  | |  | |  | |  | |  | |  | |  | |  | |  | |  |  |  |  |  |  |  |  |  |  |
| Medicare | | 4,611 | | (73.9%) | | 44,456 | | (49.8%) | | 0.514 ‡ | |  | | 6,449 | | (81.9%) | | 16,542 | | (69.9%) | | 0.285 ‡ | |  |  |  |  |  |  |  |  |  |  |
| Commercial | | 1,612 | | (25.8%) | | 44,783 | | (50.1%) | | 0.517 ‡ | |  | | 1,386 | | (17.6%) | | 7,082 | | (29.9%) | | 0.292 ‡ | |  |  |  |  |  |  |  |  |  |  |
| Medicaid | | 14 | | (0.2%) | | 105 | | (0.1%) | | 0.026 | |  | | 36 | | (0.5%) | | 58 | | (0.2%) | | 0.036 | |  |  |  |  |  |  |  |  |  |  |
| *Prior CVD diagnoses* | |  | |  | |  | |  | |  | |  | |  | |  | |  | |  | |  | |  |  |  |  |  |  |  |  |  |  |
| Myocardial infarction, N (%) | | - | |  | | - | |  | | - | |  | | 1,707 | | (21.7%) | | 4,922 | | (20.8%) | | 0.022 | |  |  |  |  |  |  |  |  |  |  |
| Stroke, N (%) | | - | |  | | - | |  | | - | |  | | 2,986 | | (37.9%) | | 9,552 | | (40.3%) | | 0.049 | |  |  |  |  |  |  |  |  |  |  |
| Unstable angina, N (%) | | - | |  | | - | |  | | - | |  | | 1,381 | | (17.5%) | | 5,131 | | (21.7%) | | 0.104 ‡ | |  |  |  |  |  |  |  |  |  |  |
| Congestive heart failure, N (%) | | - | |  | | - | |  | | - | |  | | 5,793 | | (73.6%) | | 12,020 | | (50.8%) | | 0.485 ‡ | |  |  |  |  |  |  |  |  |  |  |
| Time from last observed CVD  event to end of baseline period,  months, mean ± SD [median] | | - | |  | | - | |  | | - | |  | | 9.0 ± 12.8 [4.3] | | | | 16.8 ± 20.0 [10.6] | | | | 0.462 ‡ | |  |  |  |  |  |  |  |  |  |  |
| Less than 1 month, N (%) | | - | |  | | - | |  | | - | |  | | 2,025 | | (25.7%) | | 2,776 | | (11.7%) | | 0.365 ‡ | |  |  |  |  |  |  |  |  |  |  |
| 1 to 3 months, N (%) | | - | |  | | - | |  | | - | |  | | 1,371 | | (17.4%) | | 2,737 | | (11.6%) | | 0.167 ‡ | |  |  |  |  |  |  |  |  |  |  |
| 3 to 6 months, N (%) | | - | |  | | - | |  | | - | |  | | 1,106 | | (14.1%) | | 2,957 | | (12.5%) | | 0.046 | |  |  |  |  |  |  |  |  |  |  |
| 6 to 12 months, N (%) | | - | |  | | - | |  | | - | |  | | 1,582 | | (20.1%) | | 5,521 | | (23.3%) | | 0.078 | |  |  |  |  |  |  |  |  |  |  |
| 12 months and above, N (%) | | - | |  | | - | |  | | - | |  | | 1,787 | | (22.7%) | | 9,691 | | (40.9%) | | 0.399 ‡ | |  |  |  |  |  |  |  |  |  |  |
| Other CVD-related conditions (i.e.,  conditions used to define  CVD-related death) | | 3,133 | | (50.2%) | | 26,062 | | (29.2%) | | 0.441 ‡ | |  | | 6,911 | | (87.8%) | | 18,356 | | (77.5%) | | 0.274 ‡ | |  |  |  |  |  |  |  |  |  |  |
| *Diabetes-related characteristics* | |  | |  | |  | |  | |  | |  | |  | |  | |  | |  | |  | |  |  |  |  |  |  |  |  |  |  |
| Time from first observed  type 2 diabetes diagnosis to end  of baseline period, months,  mean ± SD [median] | | 30.9 ± 21.8 [22.9] | | | | 32.0 ± 24.6 [21.8] | | | | 0.045 | |  | | 36.6 ± 25.1 [28.2] | | | | 41.4 ± 29.4 [31.2] | | | | 0.175 ‡ | |  |  |  |  |  |  |  |  |  |  |
| Less than 12 months, N (%) | | 1,162 | | (18.6%) | | 19,003 | | (21.3%) | | 0.066 | |  | | 1,088 | | (13.8%) | | 3,165 | | (13.4%) | | 0.013 | |  |  |  |  |  |  |  |  |  |  |
| 12 to 24 months, N (%) | | 2,129 | | (34.1%) | | 29,274 | | (32.8%) | | 0.029 | |  | | 2,288 | | (29.1%) | | 6,229 | | (26.3%) | | 0.062 | |  |  |  |  |  |  |  |  |  |  |
| 24 to 48 months, N (%) | | 1,740 | | (27.9%) | | 22,653 | | (25.4%) | | 0.058 | |  | | 2,306 | | (29.3%) | | 6,300 | | (26.6%) | | 0.060 | |  |  |  |  |  |  |  |  |  |  |
| 48 months and above, N (%) | | 1,206 | | (19.3%) | | 18,414 | | (20.6%) | | 0.032 | |  | | 2,189 | | (27.8%) | | 7,988 | | (33.7%) | | 0.129 ‡ | |  |  |  |  |  |  |  |  |  |  |
| At least 1 diabetes-related hospitalization, N (%) | | 1,583 | | (25.4%) | | 16,002 | | (17.9%) | | 0.182 ‡ | |  | | 4,146 | | (52.7%) | | 9,821 | | (41.5%) | | 0.226 ‡ | |  |  |  |  |  |  |  |  |  |  |
| More than 2 diabetes-related outpatient visits, N (%) | | 5,524 | | (88.6%) | | 78,902 | | (88.3%) | | 0.008 | |  | | 7,013 | | (89.1%) | | 21,351 | | (90.2%) | | 0.035 | |  |  |  |  |  |  |  |  |  |  |
| *Charlson comorbidity index, mean ±*  *SD [median]* | | 1.5 ± 1.7 [1.0] | | | | 1.0 ± 1.4 [0.0] | | | | 0.302 ‡ | |  | | 3.5 ± 2.3 [3.0] | | | | 2.5 ± 2.2 [2.0] | | | | 0.443 ‡ | |  |  |  |  |  |  |  |  |  |  |
| 0, N (%) | | 2,260 | | (36.2%) | | 45,384 | | (50.8%) | | 0.297 ‡ | |  | | 768 | | (9.8%) | | 4,954 | | (20.9%) | | 0.314 ‡ | |  |  |  |  |  |  |  |  |  |  |
| 1, N (%) | | 1,603 | | (25.7%) | | 20,741 | | (23.2%) | | 0.058 | |  | | 753 | | (9.6%) | | 3,812 | | (16.1%) | | 0.196 ‡ | |  |  |  |  |  |  |  |  |  |  |
| 2, N (%) | | 1,135 | | (18.2%) | | 13,117 | | (14.7%) | | 0.095 | |  | | 1,184 | | (15.0%) | | 4,498 | | (19.0%) | | 0.105 ‡ | |  |  |  |  |  |  |  |  |  |  |
| 3, N (%) | | 608 | | (9.7%) | | 5,183 | | (5.8%) | | 0.148 ‡ | |  | | 1,504 | | (19.1%) | | 3,789 | | (16.0%) | | 0.082 | |  |  |  |  |  |  |  |  |  |  |
| 4 and more, N (%) | | 631 | | (10.1%) | | 4,919 | | (5.5%) | | 0.172 ‡ | |  | | 3,662 | | (46.5%) | | 6,629 | | (28.0%) | | 0.391 ‡ | |  |  |  |  |  |  |  |  |  |  |
| *Adapted diabetes complications*  *severity index, mean ± SD [median]* | | 1.9 ± 1.8 [2.0] | | | | 1.1 ± 1.4 [1.0] | | | | 0.499 ‡ | |  | | 4.1 ± 2.1 [4.0] | | | | 3.2 ± 2.0 [3.0] | | | | 0.442 ‡ | |  |  |  |  |  |  |  |  |  |  |
| 0, N (%) | | 1,833 | | (29.4%) | | 43,993 | | (49.2%) | | 0.415 ‡ | |  | | 252 | | (3.2%) | | 2,086 | | (8.8%) | | 0.238 ‡ | |  |  |  |  |  |  |  |  |  |  |
| 1, N (%) | | 1,155 | | (18.5%) | | 18,196 | | (20.4%) | | 0.047 | |  | | 327 | | (4.2%) | | 2,565 | | (10.8%) | | 0.256 ‡ | |  |  |  |  |  |  |  |  |  |  |
| 2, N (%) | | 1,292 | | (20.7%) | | 13,686 | | (15.3%) | | 0.141 ‡ | |  | | 1,520 | | (19.3%) | | 5,563 | | (23.5%) | | 0.102 ‡ | |  |  |  |  |  |  |  |  |  |  |
| 3, N (%) | | 758 | | (12.2%) | | 6,911 | | (7.7%) | | 0.148 ‡ | |  | | 1,010 | | (12.8%) | | 3,552 | | (15.0%) | | 0.063 | |  |  |  |  |  |  |  |  |  |  |
| 4 and more, N (%) | | 1,199 | | (19.2%) | | 6,558 | | (7.3%) | | 0.356 ‡ | |  | | 4,762 | | (60.5%) | | 9,916 | | (41.9%) | | 0.379 ‡ | |  |  |  |  |  |  |  |  |  |  |
| *Other recorded diagnoses, N (%)* | |  | |  | |  | |  | |  | |  | |  | |  | |  | |  | |  | |  |  |  |  |  |  |  |  |  |  |
| Hypertension | | 5,412 | | (86.8%) | | 72,289 | | (80.9%) | | 0.160 ‡ | |  | | 7,385 | | (93.8%) | | 21,687 | | (91.6%) | | 0.087 | |  |  |  |  |  |  |  |  |  |  |
| Hyperlipidemia | | 4,802 | | (77.0%) | | 71,126 | | (79.6%) | | 0.064 | |  | | 6,065 | | (77.1%) | | 19,297 | | (81.5%) | | 0.109 ‡ | |  |  |  |  |  |  |  |  |  |  |
| Infection | | 3,354 | | (53.8%) | | 43,615 | | (48.8%) | | 0.099 | |  | | 5,435 | | (69.1%) | | 14,570 | | (61.5%) | | 0.159 ‡ | |  |  |  |  |  |  |  |  |  |  |
| Mental disorders | | 1,646 | | (26.4%) | | 22,663 | | (25.4%) | | 0.023 | |  | | 3,312 | | (42.1%) | | 9,016 | | (38.1%) | | 0.082 | |  |  |  |  |  |  |  |  |  |  |
| Chronic pulmonary disease | | 1,417 | | (22.7%) | | 13,944 | | (15.6%) | | 0.181 ‡ | |  | | 3,502 | | (44.5%) | | 7,440 | | (31.4%) | | 0.272 ‡ | |  |  |  |  |  |  |  |  |  |  |
| Obesity | | 685 | | (11.0%) | | 13,609 | | (15.2%) | | 0.126 ‡ | |  | | 1,153 | | (14.6%) | | 3,998 | | (16.9%) | | 0.061 | |  |  |  |  |  |  |  |  |  |  |
| Renal failure | | 1,172 | | (18.8%) | | 9,114 | | (10.2%) | | 0.246 ‡ | |  | | 3,101 | | (39.4%) | | 6,137 | | (25.9%) | | 0.291 ‡ | |  |  |  |  |  |  |  |  |  |  |
| Cancer | | 884 | | (14.2%) | | 8,894 | | (10.0%) | | 0.130 ‡ | |  | | 1,258 | | (16.0%) | | 3,056 | | (12.9%) | | 0.088 | |  |  |  |  |  |  |  |  |  |  |
| Peripheral vascular disorders | | 1,183 | | (19.0%) | | 8,318 | | (9.3%) | | 0.280 ‡ | |  | | 2,702 | | (34.3%) | | 6,183 | | (26.1%) | | 0.180 ‡ | |  |  |  |  |  |  |  |  |  |  |
| Fluid and electrolyte disorders | | 821 | | (13.2%) | | 6,776 | | (7.6%) | | 0.184 ‡ | |  | | 2,533 | | (32.2%) | | 5,078 | | (21.4%) | | 0.244 ‡ | |  |  |  |  |  |  |  |  |  |  |
| Smoking | | 566 | | (9.1%) | | 6,828 | | (7.6%) | | 0.052 | |  | | 801 | | (10.2%) | | 2,646 | | (11.2%) | | 0.032 | |  |  |  |  |  |  |  |  |  |  |
| Deficiency anemia | | 535 | | (8.6%) | | 5,225 | | (5.8%) | | 0.106 ‡ | |  | | 1,450 | | (18.4%) | | 3,041 | | (12.8%) | | 0.154 ‡ | |  |  |  |  |  |  |  |  |  |  |
| Neuropathy/peripheral neuropathy | | 379 | | (6.1%) | | 4,057 | | (4.5%) | | 0.069 | |  | | 741 | | (9.4%) | | 1,912 | | (8.1%) | | 0.047 | |  |  |  |  |  |  |  |  |  |  |
| Rheumatoid arthritis/collagen  vascular diseases | | 336 | | (5.4%) | | 4,145 | | (4.6%) | | 0.034 | |  | | 552 | | (7.0%) | | 1,567 | | (6.6%) | | 0.016 | |  |  |  |  |  |  |  |  |  |  |
| Erectile dysfunction, organic origin | | 228 | | (3.7%) | | 3,483 | | (3.9%) | | 0.013 | |  | | 175 | | (2.2%) | | 752 | | (3.2%) | | 0.059 | |  |  |  |  |  |  |  |  |  |  |
| Coagulopathy | | 247 | | (4.0%) | | 1,868 | | (2.1%) | | 0.109 ‡ | |  | | 736 | | (9.4%) | | 1,470 | | (6.2%) | | 0.118 ‡ | |  |  |  |  |  |  |  |  |  |  |
| Phlebitis and thrombophlebitis  and other venous embolism/thrombosis | | 222 | | (3.6%) | | 1,742 | | (1.9%) | | 0.098 | |  | | 618 | | (7.9%) | | 1,267 | | (5.4%) | | 0.101 ‡ | |  |  |  |  |  |  |  |  |  |  |
| Pulmonary circulation disorder | | 213 | | (3.4%) | | 1,325 | | (1.5%) | | 0.125 ‡ | |  | | 1,134 | | (14.4%) | | 1,854 | | (7.8%) | | 0.210 ‡ | |  |  |  |  |  |  |  |  |  |  |
| Orthostatic hypotension | | 217 | | (3.5%) | | 1,528 | | (1.7%) | | 0.111 ‡ | |  | | 954 | | (12.1%) | | 1,847 | | (7.8%) | | 0.145 ‡ | |  |  |  |  |  |  |  |  |  |  |
| Alcohol abuse | | 78 | | (1.3%) | | 943 | | (1.1%) | | 0.018 | |  | | 154 | | (2.0%) | | 408 | | (1.7%) | | 0.017 | |  |  |  |  |  |  |  |  |  |  |
| Drug abuse | | 73 | | (1.2%) | | 881 | | (1.0%) | | 0.018 | |  | | 175 | | (2.2%) | | 439 | | (1.9%) | | 0.026 | |  |  |  |  |  |  |  |  |  |  |
| Peptic ulcer disease | | 69 | | (1.1%) | | 730 | | (0.8%) | | 0.030 | |  | | 181 | | (2.3%) | | 379 | | (1.6%) | | 0.051 | |  |  |  |  |  |  |  |  |  |  |

**Abbreviation:** CVD: Cardiovascular disease; MACE: Major adverse cardiovascular events; SD: Standard deviation; Std. diff: Standardized difference

‡ Indicates a standardized difference > 0.1.

[1] Any events among non-fatal myocardial infarction, non-fatal stroke, hospitalization for unstable angina, hospitalization for congestive heart failure, or CVD-related death (i.e., MACE-plus).

[2] In the primary prevention population, there were 6,237 MACE-plus (6.5%), 4,535 MACE (4.7%), and 1,694 CVD-related deaths (1.8%).

[3] In the secondary prevention population, there were 7,871 MACE-plus (24.9%), 5,199 MACE (16.5), and 2,598 CVD-related deaths (8.2%).
